# Enhancing the Electricity Generation and Nitrate Removal of Microbial Fuel Cells With a Novel Denitrifying Exoelectrogenic Strain EB-1

**DOI:** 10.3389/fmicb.2018.02633

**Published:** 2018-11-09

**Authors:** Xiaojun Jin, Fei Guo, Zhimei Liu, Yuan Liu, Hong Liu

**Affiliations:** ^1^Chongqing Institute of Green and Intelligent Technology, Chinese Academy of Sciences, Chongqing, China; ^2^University of Chinese Academy of Sciences, Beijing, China; ^3^Key Laboratory for Water Quality and Conservation of the Pearl River Delta, Ministry of Education, Institute of Environmental Research at Greater Bay, Guangzhou University, Guangzhou, China

**Keywords:** denitrifying exoelectrogens, *Mycobacterium* sp., microbial fuel cells, electron transfer, low COD/N ratios, organic materials

## Abstract

Microbial fuel cells (MFCs) have been tentatively applied for wastewater treatment, but the presence of nitrogen, especially nitrate, induces performance instability by changing the composition of functional biofilms. A novel denitrifying exoelectrogenic strain EB-1, capable of simultaneous denitrification and electricity generation and affiliated with *Mycobacterium* sp., was isolated from the anodic biofilm of MFCs fed with nitrate containing medium. Polarization curves and cyclic voltammetry showed that strain EB-1 could generate electricity through a direct electron transfer mechanism with a maximum power density of 0.84 ± 0.05 W m^−2^. Additionally, anodic denitrification, as a concurrent metabolism, was demonstrated with an efficient removal rate of 0.66 ± 0.01 kg N m^−3^ d^−1^ at a COD/N ratio of 3.5 ± 0.3. Importantly, voltage output was not negatively influenced by nitrate, indicating that the concurrent process of nitrate removal and electricity generation was a limitation of the electron donor rather than an inhibition of the system. Furthermore, various organic materials were successfully utilized as anode donors for strain EB-1, and demonstrated the exciting performances in terms of simultaneous denitrification and electricity generation. *Mycobacterium* sp. EB-1 thus expands the diversity of exoelectrogens and contributes to the potential applications of MFC for simultaneous energy recovery and wastewater treatment.

## Introduction

During the past two decades, microbial fuel cells (MFCs) have drawn global attention since they exhibit a high potential for pollutant removal and power generation. In MFCs, electrogenic bacteria oxidize organic pollutants and release electrons to the anode. Then electrons flow through the external circuit to the cathode, react with certain acceptors, and generate electricity. (Wang H. M. et al., 2015).

Wastewater treatments using MFCs mainly focus on organics removal; however, nitrogen is also present in wastewater and poses a considerable threat to lakes and other natural water bodies. Therefore, it is necessary to construct an MFC for simultaneous removal of organics and nitrogen. Recently, efficient nitrogen removal has been achieved at both the biocathode of dual-chamber MFCs (Virdis et al., 2010; Mook et al., 2013; Sevda and Sreekrishnan, 2014) and single-chamber membraneless MFCs (Yan et al., 2012; Zhang et al., 2013; Wang Z. J. et al., 2015). Nevertheless, studies on nitrate reduction (anodic denitrification) in MFCs are scarce. For example, the feasibility of coupling anodic denitrification with electricity production in MFCs was demonstrated, and anodic biofilms were found to be predominated by a significant number of nitrate-reducing microorganisms (denitrifying bacterium) (Huang et al., 2018). Therefore, it is important to study whether exoelectrogens possess the ability of denitrification simultaneously with anode respiration (where intracellular electrons are transferred to the anode by bacteria and later flow through the external circuit to the cathode for electricity generation) in MFCs and determine the reaction mechanisms in the presence of nitrate.

To date, the effect of nitrate on electricity generation by exoelectrogens has not been determined. Few studies have reported that denitrifying exoelectrogens conduct denitrification as a facultative metabolism in bioelectrochemical systems (BESs) (Xing et al., 2010; Fu et al., 2013; Kashima and Regan, 2015). The power output of MFCs with *Comamonas denitrificans* (Xing et al., 2010) or *Calditerrivibrio nitroreducens* (Fu et al., 2013) was found to be decreased when nitrate was added to the anode chamber of MFCs, suggesting that the change in metabolic pathway affected the electricity performance negatively. Kashima and Regan (2015) subsequently reported that anodic denitrification and anode respiration shifted rapidly according to nitrate concentration in a BES inoculated with the anode-respiring bacterium *Geobacter metallireducens*, and that the current output of an MFC was strongly inhibited when the nitrate concentration was above the critical level (0–56 mg N L^−1^). The critical nitrate concentrations have a significant relationship with the biofilm protein concentrations of anode biofilms, without being appreciably impacted by the anode potential. When anode biofilms were exposed to nitrate concentrations less than the critical level, simultaneous denitrification and anode respiration were observed. Besides the mentioned exoelectrogens, other denitrifying exoelectrogens such as *Shewanella oneidensis* (Cruz-Garcia et al., 2007), *Pseudomonas aeruginosa* (Manogari and Daniel, 2015; Nor et al., 2015) and *Ochrobactrum anthropic* (Zuo et al., 2008) also possess the ability of denitrification, but their performance in power generation is unclear.

Theoretically, an MFC with denitrifying exoelectrogens can perform simultaneous denitrification and electricity generation, because facultative denitrifying exoelectrogens possess two independent electron transfer mechanisms conducted by different cytochromes located in the cytoplasmic and outer membranes, catalyzing denitrification and extracellular electron transfer, respectively (Korner and Zumft, 1989; Khan and Sarkar, 2012; Kumar et al., 2016; Rajmohan et al., 2016). Furthermore, the conditions of the anode chamber satisfy the concurrent denitrification and anode respiration requirements of denitrifying exoelectrogens, including an electron donor, anoxic environment, and nitrate/electrode as an electron acceptor. Thus, denitrifying exoelectrogens could enable denitrification in the presence of nitrate, achieving simultaneous denitrification and anode respiration. However, reports have shown that low COD/N ratios significantly inhibit current generation, with no current output in MFCs (Xing et al., 2010; Fu et al., 2013; Kashima and Regan, 2015). Therefore, more studies are needed to elucidate the correlation between denitrification and anode respiration in MFC systems.

In this study, a denitrifying exoelectrogenic bacterium was isolated and identified based on 16 S rDNA gene sequencing and physiological and biochemical characterization. The electron transfer mechanism of an MFC with the isolated strain was analyzed by cyclic voltammetry (CV). The concentrations of ${\text{NO}}_{3}^{-}$-N were increased gradually from around 20 to 200 mg L^−1^ to investigate the limitation of electron donors. Afterwards, electron consumption by denitrification and anode respiration was analyzed to characterize the anodic reactions in the denitrifying MFC. Furthermore, the electron flux was discussed to illuminate the mechanisms of concurrent anode respiration and denitrification. In addition, the effects of carbon sources on electricity generation and denitrification were investigated to simulate application in wastewater treatment.

## Materials and methods

### MFC fabrication and operation

A two-chambered MFC with an air cathode was constructed to evaluate the performance of the isolated strain (Supplementary Figure S1). The two chambers were separated by a cation exchange membrane (CEM Ultrex CMI-7000), and each chamber had a volume of 15 mL. In the anodic chamber, an ammonia gas pre-treated carbon fiber felt with a volume of 2 cm^3^ (2 × 2 × 0.5 cm) was used as the anode. A carbon cloth with a surface area of 7 cm^2^ was used as the cathode and was coated with 0.5 mg cm^−2^ Pt catalyst and four layers of poly(dimethylsiloxane) on the sides facing the solution and air, respectively (Liu Y. et al., 2016). The assembled MFC was sterilized with ethyl alcohol (70%) and dried in a hot air over for 2 h at 100°C, followed by irradiation with UV light for 1 h before inoculation (Kaushik and Jadhav, 2017). The anode and cathode were connected to an external resistor of 1 kΩ via a titanium wire, and all the exposed metal surfaces were sealed with nonconductive epoxy. The voltage across the external resistor was automatically recorded every 5 min using a 64-channel data acquisition system (PCI-1747U, Advantech Co., Ltd., China) connected to a personal computer via a PCI interface (Liu et al., 2015).

Isolation of microorganisms was conducted by dilution plating on tryptic soy broth (TSB) agar plates (Chen et al., 2016) and mineral salt medium (MSM) agar plates. The MSM used as the anolyte in the MFC comprised sodium acetate, 0.1 M phosphate buffer solution (PBS, pH 7.0), KCl 0.13 g L^−1^, and Wolfe's mineral solution 12.5 mL L^−1^. Unless otherwise stated, the concentrations of sodium acetate in the MSM agar plate and liquid medium were 2 g L^−1^ and 1 g L^−1^, respectively. Nitrate concentrations were changed according to the experiment design. MSM was purged with nitrogen gas before it was sterilized to maintain an anaerobic condition and used as the anolyte, and 100 mM sterilized PBS was used as the catholyte. All of the reactors were operated in batch feed mode and were placed in an incubator with a controlled temperature of 30 ± 1°C. The anolyte was replaced with fresh MSM when the voltage decreased to below 20 mV. Two individual MFCs with strain EB-1 were operated as parallel experiments. All tests were conducted in triplicate except for CV analysis.

### Acclimatization, isolation, and identification of denitrifying exoelectrogens

The anode compartments of MFCs were firstly inoculated with a mixture (5:1 volume) of inoculum and synthetic wastewater (containing 0.72 g L^−1^ potassium nitrate) for enrichment and acclimatization of denitrifying exoelectrogens. The inoculum was the sediment from a heavily-polluted river in Chongqing. Once steady voltages were reached over several cycles, the nitrogen tests (including ${\text{NO}}_{3}^{-}$-N, ${\text{NO}}_{2}^{-}$-N, and ${\text{NH}}_{4}^{+}$-N) were conducted at the end of each cycle. Until no nitrogen was detected, isolation was performed based on dilution plating and exposure to aerobic conditions for selecting facultative anaerobic strains (Xing et al., 2010). Several colonies with different characteristics were picked up from the TSB agar plate and inoculated into a fresh TSB agar plate. The plate was then placed in an anaerobic culture box until new colonies were observed. A replicate operation was conducted with cultivation on a MSM agar plate. Colonies that could grow in both aerobic and anaerobic conditions were selected for further isolation and characterization. In the end, the obtained pure isolates were inoculated into MSM with 1 g L^−1^ sodium acetate and 2 g L^−1^ potassium nitrate to observe the growth of the pure culture under anaerobic conditions This procedure was repeated until a pure culture was obtained.

The genomic DNA of the isolated strain was extracted using an Ezup pillar bacterial genome DNA extraction kit (Shanghai Sangon, China) according to the manufacturer's instructions. The 16S rDNA gene was amplified by PCR using universal primers 27F (5′- AGAGTTTGATCCTGGCTCAG-3′) and 1492R (5′-CGGYTACCTTGTTACGACTT-3′). PCR amplification and product purification were performed according to a previous report (Deng et al., 2015). The 16S rDNA gene sequence was obtained and compared with the most related strains in the GenBank database using BLAST. Phylogenetic analysis was conducted in MEGA 5.0 software by the neighbor-joining method with the Jukes-Cantor correction (Sacco et al., 2017). A bootstrap resampling analysis was based on 1,000 replicates.

### Electrochemical analysis

To evaluate the electricity generating ability of the isolated strain, voltage outputs were collected. Furthermore, power densities and current densities were normalized to the cathode surface area. The polarization curve and power density were obtained by varying the external resistance from 100 to 5,000 Ω, collecting the voltage at each resistance when the MFC reached a steady state. The electrochemical activity of the isolated strain was detected by CV on a CHI 760E workstation (Shanghai Chenhua Instruments, China). A conventional three-electrode system was used. The anode and cathode were used as the working electrode and counter electrode, respectively, and sterilized Ag/AgCl as the reference electrodes were inserted into the anode chambers. Potential sweep experiments were performed at a scan rate of 1 mV s^−1^ from −0.6 to 0.2 V (vs. Ag/AgCl) (Fu et al., 2013). The seventh batch of MFCs operation was divided into five stages for CV analysis: (1) an initial increasing stage right after the complete replacement with fresh medium, (2) the maximum current density stage, (3) the middle point of current densities obtained in the plateau stage, (4) the decreasing stage, and (5) the end of the cycle before medium replacement.

### Morphological analysis by electron microscopy

The morphology of strain EB-1 attached to the anode surface was observed using a scanning electron microscope (JSM-6510LV, JEOL Co., Ltd., Japan). Samples were fixed with 2.5% glutaraldehyde for 4 h at room temperature, washed thrice with PBS (10 min/time) and dehydrated with a graded ethanol series (30, 50, 70, 80, and 90%; at 15 min stages). Next, the electrode pieces were dehydrated twice with 100% ethanol. The samples were finally coated with gold and observed using SEM (Zuo et al., 2008).

### Calculation

The nitrate-reducing activity of strain EB-1 was first verified by the nitrate removal rate with initial concentrations of 20, 50, 100, and 200 mg L-1 (calculated as ${\text{NO}}_{3}^{-}$-N). The acetate level remained at a theoretical COD concentration of 780.5 mg L-1. Next, samples for nitrate nitrogen (${\text{NO}}_{3}^{-}$-N), nitrite nitrogen (${\text{NO}}_{2}^{-}$-N), ammonia nitrogen (${\text{NH}}_{4}^{+}$-N) and total nitrogen were analyzed according to the Standard Methods for the Examination of Water and Wastewater using ultraviolet spectrophotometry (TU-1901, PGeneral Co.,Ltd, China).

Based on the aforementioned experiments, electron fluxes in the anode chamber were investigated in the presence of nitrate at different levels. First, electrons are produced from acetate oxidation as per equation (1) called C_t_; this production was calculated by the change in COD over time. Next, the investigation of electron fluxes in the anode chamber was divided into three parts: (i) C_an_: electrons for anode respiration were calculated by the current production over time. (ii) C_de_: electrons for anodic denitrification were calculated by assuming nitrate reduction to N_2_ from the loss of nitrate as per equation (2). In our study, nitrite accumulation of was only examined during the reduction process, whereas it disappeared at the end of the batch experiments. (iii) C_ot_: the residual part of the electrons for biomass synthesis and the electrons lost for the overpotential was calculated as: C_ot_ = C_t_-C_an_-C_de_. The coulombic efficiency (CE) of the MFC was calculated as: CE = C_an_/C_t_×100%. The corrected coulombic efficiency (CCE) excluding the electron consumption for denitrification was calculated as: CCE = C_an_/(C_t_-C_de_) × 100%. According to the reaction equation of heterotrophic denitrifiers described as equation (3), the critical value of COD/N was calculated based on the theoretical value of per gram sodium acetate transfer into 780.5 mg COD.

(1) $$\begin{array}{l} CH_{3}COO^{-}+2H_{2}O={2CO}_{2}+8e^{-}+7H^{+} \end{array}$$

(2) $$\begin{array}{l} NO_{3}^{-}+5e^{-}+6H^{+}={\frac{1}{2}N}_{2}+3H_{2}O \end{array}$$

(3) $$\begin{array}{l} 7.03\;{CH}_{3}{COO}^{-}+8.58\;{NO}_{3}^{-}=0.58C_{5}H_{7}O_{2}N\\ +11.16\;{CO}_{2}+8.58{OH}^{-}+7.74H_{2}O+4N_{2}. \end{array}$$

## Results and discussions

### Isolation and identification of strain EB-1

**Figure 1:**
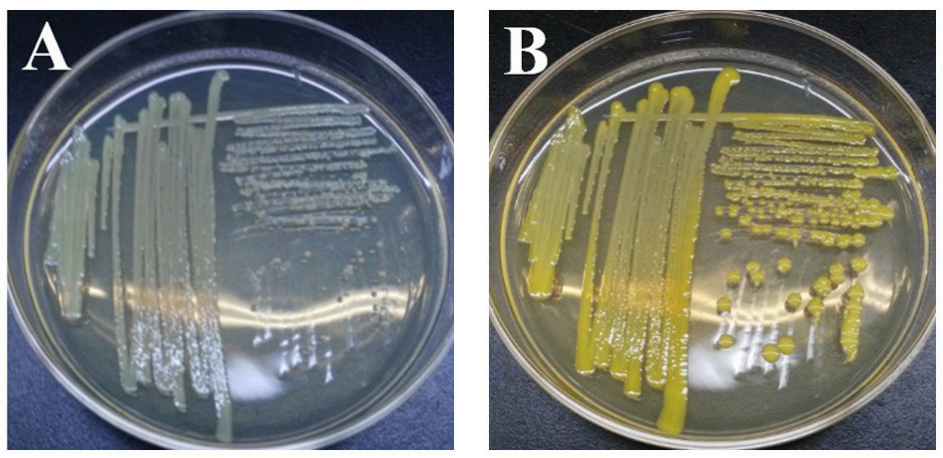
Morphology and color of strain EB-1. Picture of colonies after 48 h incubation **(A)** and pigment expression after 24 h light exposure on TSB agar medium **(B)**.

**Figure 2:**
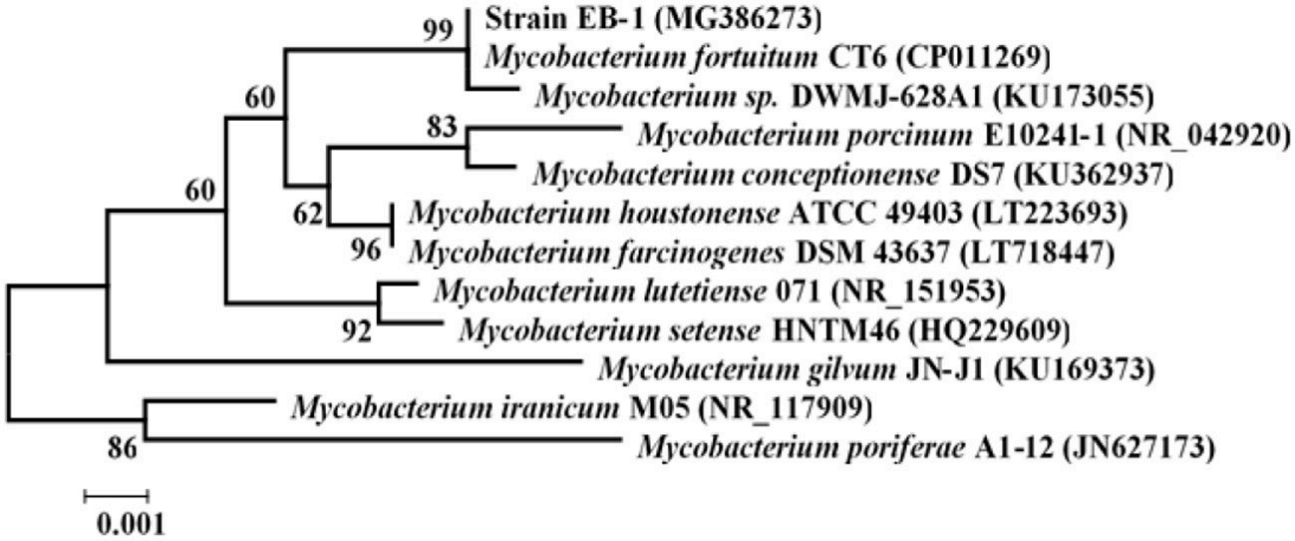
Phylogenetic tree of strain EB-1 and closely related species based on the 16S rDNA gene. The tree was constructed using the neighbor-joining method. The bootstrap values at nodes were calculated using 1,000 replicates (only values >50% are indicated); Scale Bar = 0.1% divergence.

Once exoelectrogenic denitrifying biofilms (EDBs) were enriched from the anode of MFCs fed with acetate containing MSM, the EDBs were used to isolate denitrifying exoelectrogens using an improved method based on a dilution plating procedure (Xing et al., 2010). The single strain named EB-1 is a gram-positive, nonmotile and nitrate-reducing bacterium. The pure culture was smooth, hemispheric, and cream colored after 48 h aerobic incubation on a TSB agar plate; however, a yellow pigment was observed after 24 h light exposure (Figure 1). In the MSM agar plate, the pure culture was a small white colony. Furthermore, the strain could be grown successfully with acetate as the electron donor and nitrate as the electron acceptor after incubation for 10 days, which demonstrated much lower growth rates than with oxygen used as the electron acceptor. Strain EB-1 was further analyzed and identified using PCR and 16S rDNA sequencing. Phylogenetic analysis based on the 16S rDNA gene indicated that strain EB-1 belonged to phylum *Actinobacteria* and had the closest match to *Mycobacterium fortuitum* CT6 and *Mycobacterium sp*. DWMJ-628A1 (99.9% identity) (Figure 2).

Based on characterization of the pigment secretion, strain EB-1 was recognized as one of the photochromogens affiliated to *Mycobacterium* sp., which commonly exist in biofilms of both the anode and biocathode (Liu et al., 2014; Zhang et al., 2017). Members of *Mycobacterium* sp. are distributed widely and have been detected in natural and processed water, sewage, and sludge (Weber et al., 2000; Liu et al., 2014). Several *Mycobacterium* species (e.g., *M. tuberculosis, M. bovis*, and *M. smegmatis*) have been verified to produce anaerobic nitrate reductase, an enzyme contributing to nitrate reduction (Martin et al., 2008; Huang et al., 2015). However, the exoelectrogenic activity of *Mycobacterium* sp. has never been investigated. To our knowledge, this is the first report that *Mycobacterium* genus has the ability of extracellular electron transfer, though this genus has been widely existent in biological reactors for wastewater treatment and the biocathodes of BESs (Vilajeliu-Pons et al., 2015).

The 16S rDNA sequence of strain EB-1 determined in this study has been deposited in the GenBank database with the accession number MG386273. The isolated strain EB-1 has been stored at the China Center for Type Culture Collection under depository number CCTCC M2017371.

### Power generation and electron transfer mechanism of strain EB-1

**Figure 3:**
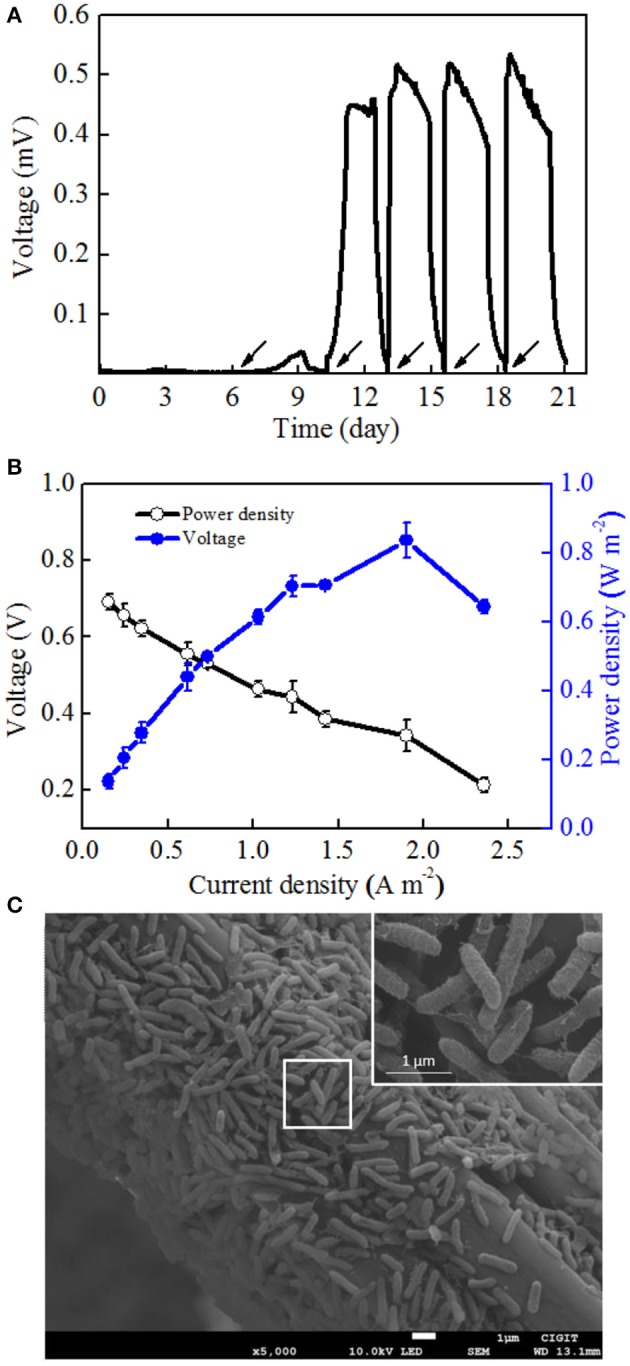
MFC performance after startup. **(A)** Voltage curves of strain EB-1using acetate sodium as an electron acceptor. **(B)** Polarization curves and power densities as a function of current densities. **(C)** SEM images of the strain EB-1 biofilm on the carbon fiber felt of MFCs. *Arrows* show the addition of acetate sodium.

To determine the electrochemical activity of *Mycobacterium* sp. EB-1, the pure culture was inoculated into MFCs. Synthetic wastewater without nitrate was added into the anodic chambers. Figure 3A shows the low voltage output in the first two cycles after startup and that the voltage was rapidly increased to 450.4 mV in the third cycle. In the following cycles, the voltage was immediately recovered to the reproducible level and reached a plateau once the anolyte was replaced with fresh medium. After four cycles of operation, stable maximum voltages could be observed. A maximum power density (MPD) of 0.84 ± 0.05 W m^−2^ was obtained at a current density of 1.9 ± 0.01 A m^−2^ (Figure 3B), which was comparable to that of other pure cultures such as *Citrobacter* sp. LAR-1 (0.61 W m^−2^) (Liu L.H. et al., 2016) and *Comamonas denitrificans* DX-4 (0.036 W m^−2^) (Xing et al., 2010).

In MFCs, exoelectrogens attached to electrodes as biofilms play a key role in current generation. Currently, the two acknowledged mechanisms of electron transfer include (i) mediated electron transfer (MET) with extracellular electron shuttle or reduced secondary metabolites secreted by bacteria, and (ii) direct electron transfer (DET) by membrane-bound c-type cytochromes and / or nanowires (or conductive pili) (Reguera et al., 2005; Xu and Liu, 2011). To date, these electron transfer mechanisms in MFCs were based on two model exoelectrogens, *Shewanella oneidensis* MR-1 and *Geobacter sulfurreducens* PCA, both gram-negative bacteria. The electron transfer mechanisms of gram-positive exoelectrogens have not been thoroughly investigated.

In this section, the electron transfer mechanism of strain EB-1 was discussed with respect to morphology and CV analysis. As illustrated in SEM images, the biofilm of strain EB-1 developed well on the anode surface (Figure 3C). Morphology analysis indicated that strain EB-1 is a rod-shaped (0.2–0.4 μm wide and 1–3 μm long) bacterium, and that a large number of nanowires touch both the surface of the anode and other cells, thus forming a nano-grid (Reguera et al., 2005). Replacement experiments also suggested that the electricity generation of strain EB-1 could be attributed to the bacteria attached to the anode rather than to soluble mediators or planktonic cells (Figure 3A).

**Figure 4:**
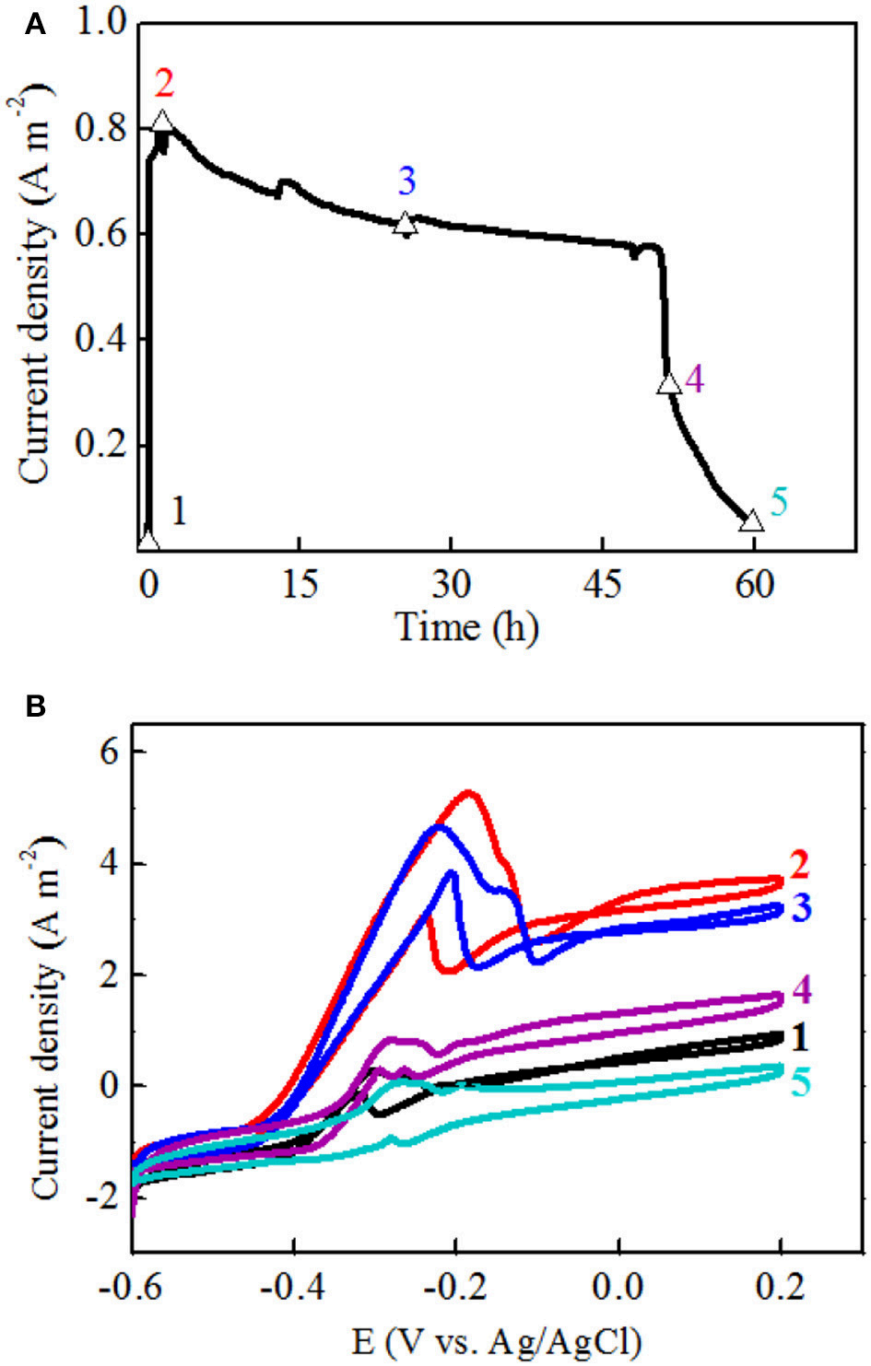
One batch of current generation divided into five stages for CV analysis **(A)**: 1. the initial increasing stage; 2. the maximum current density stage; 3. the plateau stage; 4. the decreasing stage; 5. the end of batch. CV collected at each point of one batch of the current generation **(B)**.

To further investigate the electron transfer mechanism of strain EB-1, CV profiles of the anodic biofilm at five points of one full batch were collected (Figure 4). It was supposed that no accumulation of secreted mediators was obtained for the changes in oxidation peak height corresponding to those in voltage outputs. On the contrary, if mediators had been involved in the electron transfer, the oxidation peak height of stage 3 would be higher than that of stage 2, because the production, excretion, and accumulation of these secondary metabolic products would take substantial amounts of time to reach a sufficient amount for peaks in CVs (Xu and Liu, 2011). In this study, when the anolyte was replaced with fresh medium, the current density were recovered to 41.2% and 92.1% of the maximum density within 5 min and 10 min, respectively (Figure 4A). More specifically, an increase in the oxidization peak height was observed immediately at the initial increasing stage (Supplementary Figure S2). Therefore, the rapid recovery of electrochemical activity should be attributed to the direct attachment of strain EB-1 to the anode.

**Figure 5:**
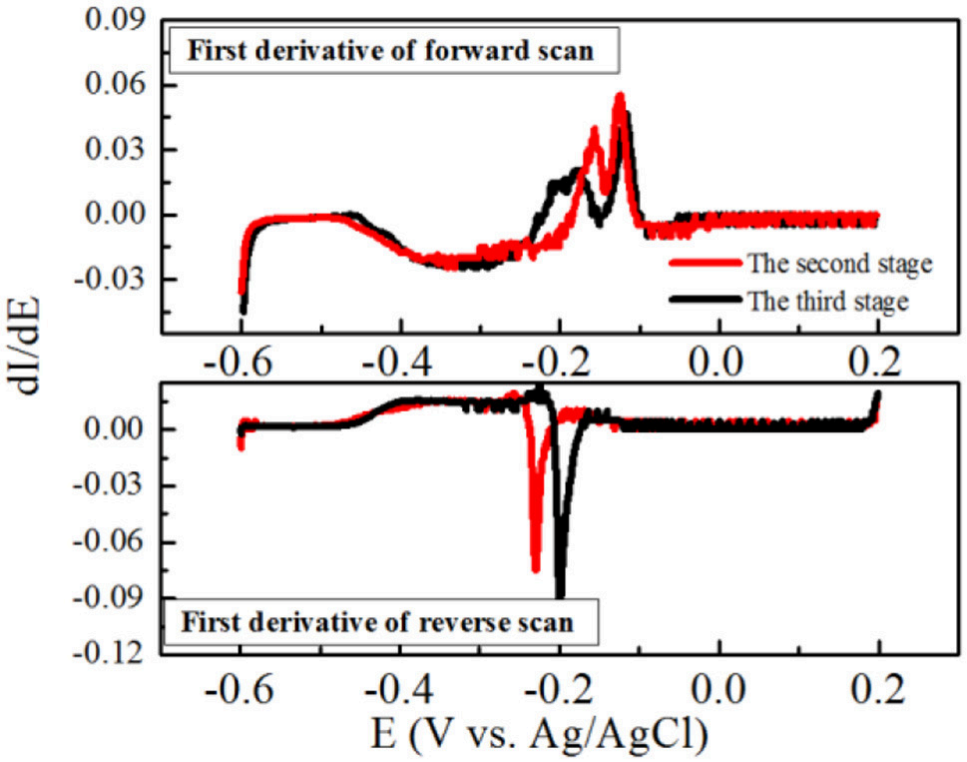
First derivatives of the voltammetric curves over the potential at the second and third stage, respectively.

Furthermore, the maximum current density was obtained at the second stage, and the obvious oxidation and reduction peak potentials of the redox pair were −0.21 V and −0.185 V (vs. Ag/AgCl electrode), respectively (Figure 4B). Another potential oxidation peak was also observed in Figure 3B, which was verified by the first derivative curve. The first derivative curve of this CV revealed that the oxidation potential sweep possesses two inflection points and only one reductive potential point (Figure 5). A similar phenomenon of CV was also obtained at the third stage. In the following stages, CV showed that the oxidation and reduction peak potentials were decreased with the decreased current densities. This strange voltammetric behavior heavily depends on the scan rate of the voltammetric experiment. The anodic electron transfer of *Geobacter sulfurreducensis* considered to be an archetype for direct electron transfer, showed one, two and four major redox systems for a scan rate of 50 mV s^−1^, 5 mV s^−1^, and 1 mV s^−1^, respectively (Fricke et al., 2008). Even regarding microbial cell membranes, bacterial cultures may contain several redox active species that do not necessarily contribute to the bioelectrocatalytic current flow. A similar phenomenon of CV was also obtained at the third stage.

This reductive potential is close to the outer membrane multiheme c-type cytochromes OmcA of −0.201 V in *Shewanella oneidensis* (Meitl et al., 2009) or the cytoplasmic membrane multiheme c-type cytochromes CymA of −0.229 V in *Shewanella frigidimarina* (Field et al., 2000). Therefore, it is suggested that electricity generation of strain EB-1 in the MFC was probably mediated by cell-bound outer membrane proteins. Overall, the electron transfer mechanism of strain EB-1 should be considered to be direct electron transfer.

### Simultaneous electricity generation and denitrification in MFCs with EB-1

**Figure 6:**
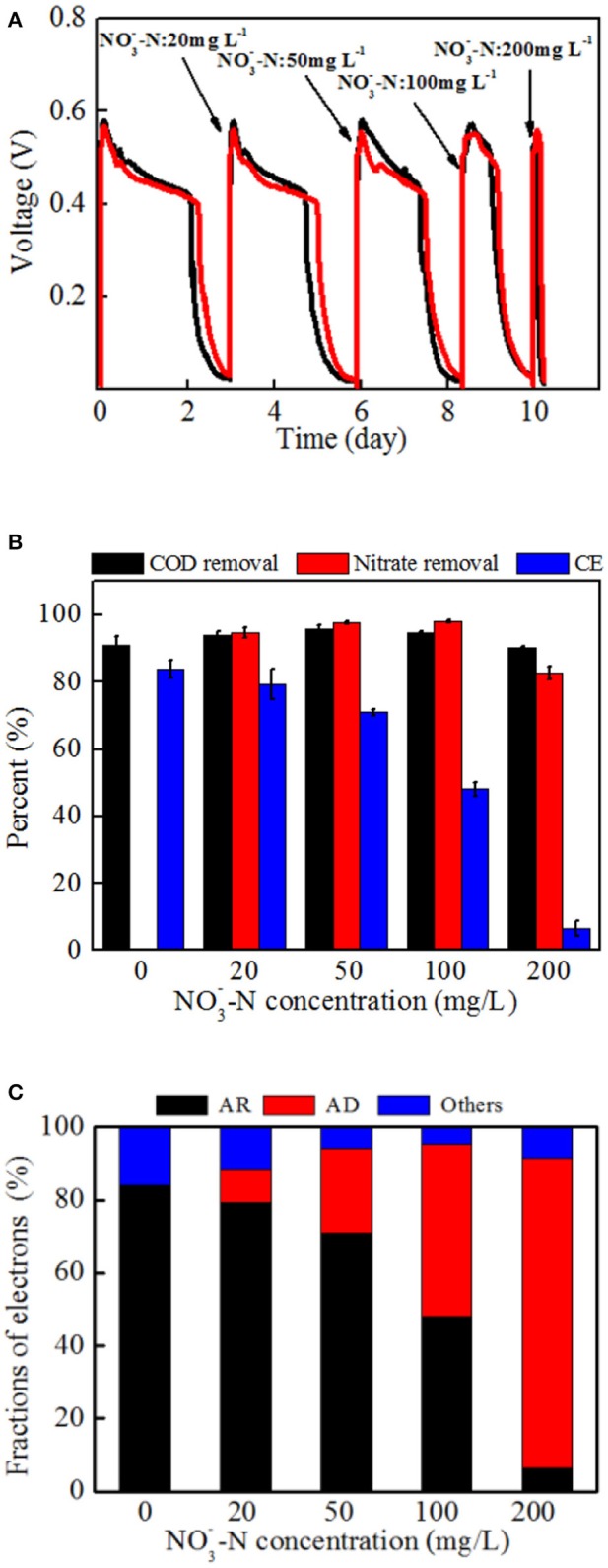
The performance and electron tendency of MFCs inoculated with strain EB-1: **(A)** Voltage outputs, **(B)** carbon and nitrate removal, and energy recovery, **(C)** electron fraction of MFCs. AR: the electrons for anode respiration; AD: the electrons for anodic denitrification; Others: electrons for biomass synthesis and electron losses for overpotential. A fixed external resistance (1 kΩ) was connected into the circuit between the anode and cathode.

Different power generation cycles of MFCs with strain EB-1 were demonstrated with ${\text{NO}}_{3}^{-}$-N concentrations ranging from 0 to 200 mg L^−1^. As shown in Figure 6A, the duration of each cycle was shortened with the increasing ${\text{NO}}_{3}^{-}$-N concentration, whereas the maximum voltage output (V_max_) was rarely affected. The concurrent processes of denitrification and anode respiration are thus a limitation of the electron donor rather than an inhibition of the system. In our system, the anode and cathode was connected with a fixed external resistance of 1 kΩ, demonstrating the limited rate of extracellular electron transfer for anode respiration. *Calditerrivibrio nitroreducens* strain Yu37-1, as a nitrate reducer, strongly inhibited current generation with 20 mM nitrate (280 mg L^−1^ ${\text{NO}}_{3}^{-}$-N) (Fu et al., 2013). The external resistance between the anode and cathode was 100 Ω. Another denitrifying exoelectrogen, *Comamonas denitrificans* DX-4, also produced decreased voltage when 10 mM nitrate was added to the anolyte of a MFC with 1 kΩ external resistance (Xing et al., 2010). Inhibition of current generation by nitrate was also confirmed in a bioelectrochemical system, whereas simultaneous anode respiration and denitrification were realized in our research. The concurrent metabolism of anode respiration and denitrification was possibly related to the electron fluxes (Fu et al., 2013; Kashima and Regan, 2015). In addition, through the domestication process, the nitrate in the anolyte induces denitrification gene expression and stimulates a series of enzymes synthesis in strain EB-1, which finally lead to denitrification in the MFC. In analogous systems, the exoelectrogen *Thermincola potens* coupled acetate oxidation to the reduction of hydrous ferric oxides in the anode chamber, showing simultaneous electricity generation and metal reduction in MFC. It is also proposed that electron transfer by cell wall-associated cytochromes and metal reduction are observed in a gram-positive bacterium (Carlson et al., 2012).

An apparent reduction of CE (from 83.9 ± 2.7% to 6.5 ± 2.2%) was observed with increasing nitrate concentration, indicating that denitrification in the anode chamber inevitably consumed more electrons from acetate oxidation. The anolyte without nitrate was operated as a control experiment and is shown in the first cycle of Figure 6A. Additionally, an anolyte without acetate was also investigated in the MFCs with voltage outputs not more than 20 mV and only 5% nitrate removal (data not shown).

As shown in Figure 6B, almost all of nitrate was removed when the ${\text{NO}}_{3}^{-}$-N concentration was below 100 mg L^−1^. Further, 82.8 ± 6.5 % ${\text{NO}}_{3}^{-}$-N was removed within the first 6 h when 200 mg L^−1^ ${\text{NO}}_{3}^{-}$-N was added to the anolyte. The nitrogen content of end-products demonstrated that nitrate was reduced to nitrogen gas and no ammonium was detected in the anolyte (Supplementary Table S1). The content of nitrite was under 1 mg L^−1^ in all tests. It is thus supposed that the denitrification in the MFC follows the first-order kinetics equation as reported previously (Drewnowski and Fernandez-Morales, 2016). An average nitrate removal rate of 0.66 ± 0.01 kg N m^−3^ d^−1^ (27.7 ± 0.5 mg N L^−1^ h^−1^) was achieved in high nitrate conditions (200 mg L^−1^ ${\text{NO}}_{3}^{-}$-N), which was faster than that observed in a biocathode MFC of 0.02–0.43 kg N m^−3^ d^−1^ (He and Angenent, 2006; Fang et al., 2011; Sotres et al., 2016). Dissimilatory nitrate reduction to ammonium is also shown to be a possible electron sink during cathodic denitrification in BESs (Sander et al., 2015). In the conventional heterotrophic denitrification for the treatment of low-COD/N domestic wastewater, the observed specific denitrification rates are 0.08–1.36 kg N m^−3^ d^−1^ with sufficient carbon source and 0.02–0.12 kg N m^−3^ d^−1^ without exogenous carbon (Sun et al., 2010; Veys et al., 2010).

**Figure 7:**
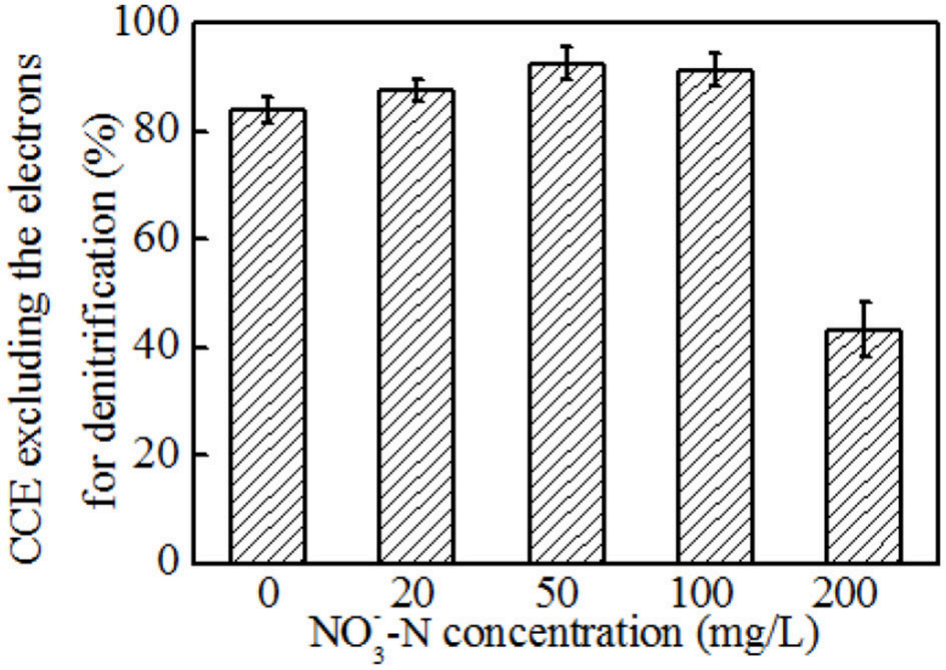
The CCEs of MFCs operated in the absence and presence of nitrate.

Based on the above analysis, the electron fluxes in the anodic chamber of the MFCs in the absence and presence of nitrate were investigated. The electron fluxes could be divided into three parts, containing the anodic denitrification, anode respiration, and others which included biomass synthesis and electron losses for overpotential. Though the electrons for anode respiration were sharply decreased with an increasing amount of nitrate introduced into the anodic chamber, the electrons for denitrification were increased (Figure 6C). Further analysis demonstrated that the CCEs increased and then decreased, and a maximum value of 92.4 ± 3.0 % was obtained when the ${\text{NO}}_{3}^{-}$-N concentration was 50 mg L^−1^ (Figure 7). The CCE sharply decreased to 43.3 ± 5.0 % at the ${\text{NO}}_{3}^{-}$-N concentration of 200 mg L^−1^. The changes in CCEs indicated that a critical condition existed for the symbiotic metabolisms of denitrification and anode respiration in MFCs inoculated with exoelectrogenic denitrifying bacteria (Kashima and Regan, 2015). In this experiment, calculation of electrons in terms of intermediates (including nitrite, nitrous oxides, and ammonium) was negligible due to concentrations under the limit of detection. Therefore, the critical value was calculated by the reduction of nitrate to N_2_. For the denitrifying exoelectrogen EB-1, the net COD/N ratios for denitrification were 9.36 ± 0.05, 3.75 ± 0.03, 3.23 ± 0.02, and 3.18 ± 0.03 at the initial ${\text{NO}}_{3}^{-}$-N concentrations of 20, 50, 100, and 200 mg L^−1^, respectively. The critical COD/N ratio of 3.75 seems to be the optimal condition for simultaneous denitrification and electricity generation in denitrifying MFCs, which was consistent with the traditional biological heterotrophic denitrifiers (BHD). According to equation 3, the critical COD/N ratio of 3.75 was calculated in BHD. In packed-bed biological reactors, a decrease in the COD/N ratio from 13.5 to 6.25 resulted in ${\text{NO}}_{2}^{-}$-N accumulation (Karanasios et al., 2016). However, no apparent ${\text{NO}}_{2}^{-}$-N accumulation was observed in our study, even when the COD/N was below 3. This indicates that simultaneous carbon and nitrate removal by denitrifying exoelectrogens in MFCs is more efficient than that by BHD in the low COD/N wastewater treatment. Furthermore, the redundant carbon sources can be used as electron donors in the MFC system, thereby recovering the energy and reducing excessive sludge production.

### Different electron donors used for strain EB-1

**Figure 8:**
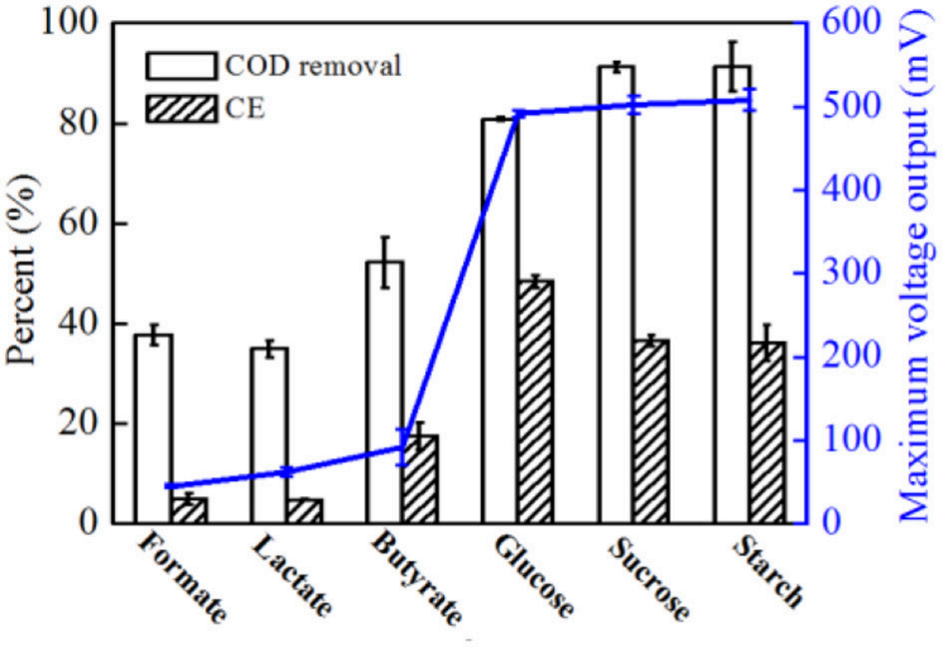
COD removal rate, CE and maximum voltage output of MFCs with different substrates. For all substrates, 500 mg L^−1^ COD and 50 mg L^−1^ ${\text{NO}}_{3}^{-}$-N were added and experiments were conducted in duplicate using three individual MFC reactors.

In addition to acetate, formate, lactate, butyrate, glucose, sucrose, and starch were also used as electron donors; however, the COD and ${\text{NO}}_{3}^{-}$-N concentrations remained at the same level for all substrates at 500 and 50 mg L-1, respectively. Higher voltage output was observed when glucose, sucrose and starch were used (Supplementary Figure S3). As shown in Figure 8, higher CEs were obtained with glucose than with sucrose and starch. Compared to acetate, much lower COD removal (< 80.8%) were observed with all substrates, indicating that with a more complex carbohydrate used by strain EB-1, less energy was recovered from the complex metabolism. Additionally, almost all of ${\text{NO}}_{3}^{-}$-N was removed by strain EB-1, indicating that a part of the electrons generated from the substrate oxidation was consumed by denitrification (Chae et al., 2009; Feng et al., 2013). These results indicate that strain EB-1 can utilize a wide range of carbon sources for electricity generation and denitrification in MFCs, which increases the potential applications of MFCs in renewable energy generation and nitrate-contaminated wastewater treatment.

## Conclusions

*Mycobacterium* sp. EB-1 was isolated and identified as a novel denitrifying exoelectrogenic bacterium based on 16S rDNA gene sequencing. CV analysis revealed that strain EB-1 was capable of producing electricity by direct electron transfer. With increased nitrate concentration, the performance of MFCs inoculated with strain EB-1 demonstrated stable denitrification and electricity generation, with no negative influence on the corresponding voltages were not d. The anodic denitrification as a concurrent metabolism was independent to electricity generation and more efficient at utilizing the limited carbon sources in low-COD/N wastewater. The wide range of substrate utilization by strain EB-1 increases the application potential of MFCs in renewable energy generation and wastewater treatment.

## Author contributions

XJJ designed the experimental protocol, carried out the majority of the experiments, and draft the manuscript. FG corrected the paper. ZML helped to copy and verify the experiments results. YL and HL supervised the study and contributed to the critical review of the manuscript. All authors read and approved the final manuscript.

### Conflict of interest statement

The authors declare that the research was conducted in the absence of any commercial or financial relationships that could be construed as a potential conflict of interest.

## References

[B1] Carlson H. K. Iavarone A. T. Gorur A. Yeo B. S. Tran R. Melnyk R. A.. (2012). Surface multiheme c-type cytochromes from Thermincola potens and implications for respiratory metal reduction by Gram- positive bacteria. P. Natl. Acad. Sci. USA. 109, 1702–1707. 10.1073/pnas.1112905109 22307634 PMC3277152

[B2] Chae K. J. Choi M. J. Lee J. W. Kim K. Y. Kim I. S. (2009). Effect of different substrates on the performance, bacterial diversity, and bacterial viability in microbial fuel cells. Bioresour. Technol. 100, 3518–3525. 10.1016/j.biortech.2009.02.065 19345574

[B3] Chen D.-Z. Jin X.-J. Chen J. Ye J.-X. Jiang N.-X. Chen J.-M. (2016). Intermediates and substrate interaction of 1,4-dioxane degradation by the effective metabolizer *Xanthobacter flavus* DT8. Int. Biodeterior. Biodegrad. 106, 133–140. 10.1016/j.ibiod.2015.09.018

[B4] Cruz-Garcia C. Murray A. E. Klappenbach J. A. Stewart V. Tiedje J. M. (2007). Respiratory nitrate ammonification by Shewanella oneidensis MR-1. J. Bacteriol. 189, 656–662. 10.1128/JB.01194-06 17098906 PMC1797406

[B5] Deng D. D. Zhang Y. C. Liu Y. (2015). A *Geobacter* strain isolated from rice paddy soil with higher bioelectricity generation capability in comparison to *Geobacter sulfurreducens* PCA. Rsc Adv. 5, 43978–43989. 10.1039/C5RA06211J

[B6] Drewnowski J. Fernandez-Morales F. J. (2016). Heterotrophic anodic denitrification in microbial fuel cells. Sustainability 8:561 10.3390/su8060561

[B7] Fang C. Min B. Angelidaki I. (2011). Nitrate as an oxidant in the cathode chamber of a microbial fuel cell for both power generation and nutrient removal purposes. Appl. Biochem. Biotechnol. 164, 464–474. 10.1007/s12010-010-9148-0 21188547

[B8] Feng H. J. Huang B. C. Zou Y. Q. Li N. Wang M. Z. Yin J.. (2013). The effect of carbon sources on nitrogen removal performance in bioelectrochemical systems. Bioresour. Technol. 128, 565–570. 10.1016/j.biortech.2012.11.004 23211481

[B9] Field S. J. Dobbin P. S. Cheesman M. R. Watmough N. J. Thomson A. J. Richardson D. J. (2000). Purification and magneto-optical spectroscopic characterization of cytoplasmic membrane and outer membrane multiheme c-type cytochromes from Shewanella frigidimarina NCIMB400. J. Biol. Chem. 275, 8515–8522. 10.1074/jbc.275.12.8515 10722689

[B10] Fricke K. Harnisch F. Schröder U. (2008). On the use of cyclic voltammetry for the study of anodic electron transfer in microbial fuel cells. Energ. Environ. Sci. 1, 144–147. 10.1039/b802363h

[B11] Fu Q. Kobayashi H. Kawaguchi H. Wakayama T. Maeda H. Sato K. (2013). A thermophilic gram-negative nitrate-reducing bacterium, calditerrivibrio nitroreducens, exhibiting electricity generation capability. Environ. Sci. Technol. 47, 12583–12590. 10.1021/es402749f 24053548

[B12] He Z. Angenent L. T. (2006). Application of bacterial biocathodes in microbial fuel cells. Electroanalysis 18, 2009–2015. 10.1002/elan.200603628

[B13] Huang H. B. Cheng S. A. Yang J. W. Li C. C. Sun Y. Cen K. F. (2018). Effect of nitrate on electricity generation in single-chamber air cathode microbial fuel cells. Chem. Eng. J. 337, 661–670. 10.1016/j.cej.2017.12.150

[B14] Huang Q. Abdalla A. E. Xie J. (2015). Phylogenomics of *Mycobacterium* nitrate reductase operon. Curr. Microbiol. 71, 121–128. 10.1007/s00284-015-0838-2 25980349

[B15] Karanasios K. A. Vasiliadou I. A. Tekerlekopoulou A. G. Akratos C. S. Pavlou S. Vayenas D. V. (2016). Effect of C/N ratio and support material on heterotrophic denitrification of potable water in bio-filters using sugar as carbon source. Int. Biodeterior. Biodegrad. 111, 62–73. 10.1016/j.ibiod.2016.04.020

[B16] Kashima H. Regan J. M. (2015). Facultative nitrate reduction by electrode-respiring *Geobacter* metallireducens biofilms as a competitive reaction to electrode reduction in a bioelectrochemical system. Environ. Sci. Technol. 49, 3195–3202. 10.1021/es504882f 25622928

[B17] Kaushik A. Jadhav S. K. (2017). Conversion of waste to electricity in a microbial fuel cell using newly identified bacteria: *Pseudomonas fluorescens*. Int. J. Environ. Sci. Technol. 14, 1771–1780. 10.1007/s13762-017-1260-z

[B18] Khan A. Sarkar D. (2012). Nitrate reduction pathways in mycobacteria and their implications during latency. Microbiology 158, 301–307. 10.1099/mic.0.054759-0 22174380

[B19] Korner H. Zumft W. G. (1989). Expression of denitrification enzymes in response to the dissolved oxygen level and respiratory substrate in continuous culture of *Pseudomonas stutzeri*. Appl. Environ. Microbiol. 55, 1670–1676. 2764573 10.1128/aem.55.7.1670-1676.1989 PMC202933

[B20] Kumar R. Singh L. Zularisam A. W. (2016). Exoelectrogens: recent advances in molecular drivers involved in extracellular electron transfer and strategies used to improve it for microbial fuel cell applications. Renew. Sust. Energy Rev. 56, 1322–1336. 10.1016/j.rser.2015.12.029

[B21] Liu G. L. Yu S. X. Luo H. P. Zhang R. D. Fu S. Y. Luo X. N. (2014). Effects of salinity anions on the anode performance in bioelectrochemical systems. Desalination 351, 77–81. 10.1016/j.desal.2014.07.026

[B22] Liu L. H. Lee D. J. Wang A. J. Ren N. Q. Su A. Lai J. Y. (2016). Isolation of Fe(III)-reducing bacterium, *Citrobacter* sp LAR-1, for startup of microbial fuel cell. Int. J. Hydrogen Energ. 41, 4498–4503. 10.1016/j.ijhydene.2015.07.072

[B23] Liu Y. Jin X. J. Dionysiou D. D. Liu H. Huang Y. M. (2015). Homogeneous deposition-assisted synthesis of iron nitrogen composites on graphene as highly efficient non-precious metal electrocatalysts for microbial fuel cell power generation. J. Power Sources 278, 773–781. 10.1016/j.jpowsour.2014.12.134

[B24] Liu Y. Jin X. J. Tuo A. X. Liu H. (2016). Improved oxygen reduction reaction activity of three-dimensional porous N-doped graphene from a soft-template synthesis strategy in microbial fuel cells. Rsc Adv. 6, 105211–105221. 10.1039/C6RA23971D

[B25] Manogari R. Daniel D. K. (2015). Isolation, characterization and assessment of *Pseudomonas* sp VITDM1 for electricity generation in a microbial fuel cell. Indian J. Microbiol. 55, 8–12. 10.1007/s12088-014-0491-7

[B26] Martin A. Panaiotov S. Portaels F. Hoffner S. Palomino J. C. Angeby K. (2008). The nitrate reductase assay for the rapid detection of isoniazid and rifampicin resistance in *Mycobacterium tuberculosis*: a systematic review and meta-analysis. J. Antimicrob. Chemother. 62, 56–64. 10.1093/jac/dkn139 18407918

[B27] Meitl L. A. Eggleston C. M. Colberg P. J. S. Khare N. Reardon C. L. Shi L. (2009). Electrochemical interaction of *Shewanella oneidensis* MR-1 and its outer membrane cytochromes OmcA and MtrC with hematite electrodes. Geochim. Cosmochim. Acta. 73, 5292–5307. 10.1016/j.gca.2009.06.021

[B28] Mook W. T. Aroua M. K. T. Chakrabarti M. H. Noor I. M. Irfan M. F. Low C. T. J. (2013). A review on the effect of bio-electrodes on denitrification and organic matter removal processes in bio-electrochemical systems. J. Ind. Eng. Chem. 19, 1–13. 10.1016/j.jiec.2012.07.004

[B29] Nor M. H. M. Mubarak M. F. M. Elmi H. S. A. Ibrahim N. Wahab M. F. A. Ibrahim Z. (2015). Bioelectricity generation in microbial fuel cell using natural microflora and isolated pure culture bacteria from anaerobic palm oil mill effluent sludge. Bioresour. Technol. 190, 458–465. 10.1016/j.biortech.2015.02.103 25799955

[B30] Rajmohan K. S. Gopinath M. Chetty R. (2016). Review on challenges and opportunities in the removal of nitrate from wastewater using electrochemical method. J. Environ. Biolog. 37, 1519–1528.

[B31] Reguera G. McCarthy K. D. Mehta T. Nicoll J. S. Tuominen M. T. Lovley D. R. (2005). Extracellular electron transfer via microbial nanowires. Nature 435, 1098–1101. 10.1038/nature03661 15973408

[B32] Sacco N. J. Bonetto M. C. Corton E. (2017). Isolation and characterization of a novel electrogenic bacterium, *Dietzia* sp RNV-4. PLoS ONE 12:e0169955. 10.1371/journal.pone.0169955 28192491 PMC5305051

[B33] Sander E. M. Virdis B. Freguia S. (2015). Dissimilatory nitrate reduction to ammonium as an electron sink during cathodic denitrification. Rsc Adv. 5, 86572–86577. 10.1039/C5RA19241B

[B34] Sevda S. Sreekrishnan T. R. (2014). Removal of organic matters and nitrogenous pollutants simultaneously from two different wastewaters using biocathode microbial fuel cell. J. Environ. Sci. Heal. A 49, 1265–1275. 10.1080/10934529.2014.910064 24967560

[B35] Sotres A. Cerrillo M. Vinas M. Bonmati A. (2016). Nitrogen removal in a two-chambered microbial fuel cell: establishment of a nitrifying-denitrifying microbial community on an intermittent aerated cathode. Chem. Eng. J. 284, 905–916. 10.1016/j.cej.2015.08.100

[B36] Sun S. P. Nacher C. P. I. Merkey B. Zhou Q. Xia S. Q. Yang D. H. (2010). Effective biological nitrogen removal treatment processes for domestic wastewaters with low C/N Ratios: a review. Environ. Eng. Sci. 27, 111–126. 10.1089/ees.2009.0100

[B37] Veys P. Vandeweyer H. Audenaert W. Monballiu A. Dejans P. Jooken E.. (2010). Performance analysis and optimization of autotrophic nitrogen removal in different reactor configurations: a modelling study. Environ. Technol. 31, 1311–1324. 10.1080/09593331003713685 21121455

[B38] Vilajeliu-Pons A. Puig S. Pous N. Salcedo-Davila I. Baneras L. Balaguer M. D.. (2015). Microbiome characterization of MFCs used for the treatment of swine manure. J Hazard. Mater. 288, 60–68. 10.1016/j.jhazmat.2015.02.014 25698567

[B39] Virdis B. Rabaey K. Rozendal R. A. Yuan Z. G. Keller J. (2010). Simultaneous nitrification, denitrification and carbon removal in microbial fuel cells. Water Res. 44, 2970–2980. 10.1016/j.watres.2010.02.022 20303136

[B40] Wang H. M. Park J. D. Ren Z. J. (2015). Practical energy harvesting for microbial fuel cells: a review. Environ. Sci. Technol. 49, 3267–3277. 10.1021/es5047765 25670167

[B41] Wang Z. J. Zhang B. G. Borthwick A. G. L. Feng C. P. Ni J. R. (2015). Utilization of single-chamber microbial fuel cells as renewable power sources for electrochemical degradation of nitrogen-containing organic compounds. Chem. Eng. J. 280, 99–105. 10.1016/j.cej.2015.06.012

[B42] Weber I. Fritz C. Ruttkowski S. Kreft A. Bange F. (2000). Anaerobic nitrate reductase (narGHJI) activity of *Mycobacterium bovis* BCG *in vitro* and its contribution to virulence in immunodeficient mice. Mol. Microbiol. 35, 1017–1025. 10.1046/j.1365-2958.2000.01794.x 10712684

[B43] Xing D. F. Cheng S. A. Logan B. E. Regan J. M. (2010). Isolation of the exoelectrogenic denitrifying bacterium *Comamonas denitrificans* based on dilution to extinction. Appl. Microbiol. Biotechnol. 85, 1575–1587. 10.1007/s00253-009-2240-0 19779712

[B44] Xu S. Liu H. (2011). New exoelectrogen *Citrobacter* sp SX-1 isolated from a microbial fuel cell. J. Appl. Microbiol. 111, 1108–1115. 10.1111/j.1365-2672.2011.05129.x 21854512

[B45] Yan H. J. Saito T. Regan J. M. (2012). Nitrogen removal in a single-chamber microbial fuel cell with nitrifying biofilm enriched at the air cathode. Water Res. 46, 2215–2224. 10.1016/j.watres.2012.01.050 22386083

[B46] Zhang G. D. Feng S. S. Jiao Y. Lee D. J. Xin Y. J. Sun H. F. (2017). Cathodic reducing bacteria of dual-chambered microbial fuel cell. Int. J. Hydrogen Energ. 42, 27607–27617. 10.1016/j.ijhydene.2017.06.095

[B47] Zhang G. Y. Zhang H. M. Zhang C. Y. Zhang G. Q. Yang F. L. Yuan G. G. (2013). Simultaneous nitrogen and carbon removal in a single chamber microbial fuel cell with a rotating biocathode. Process Biochem. 48, 893–900. 10.1016/j.procbio.2013.03.008

[B48] Zuo Y. Xing D. F. Regan J. M. Logan B. E. (2008). Isolation of the exoelectrogenic bacterium *Ochrobactrum anthropi* YZ-1 by using a U-tube microbial fuel cell. Appl. Environ. Microbiol. 74, 3130–3137. 10.1128/AEM.02732-07 18359834 PMC2394939

