# Supplementary material for: Enhancing the Electricity Generation and Nitrate Removal of Microbial Fuel Cells With a Novel Denitrifying Exoelectrogenic Strain EB-1
Source: Front Microbiol. 2018 Nov 9;9:2633. doi: 10.3389/fmicb.2018.02633 (PMC6237982; doi:10.3389/fmicb.2018.02633)
Supplement: Supplementary file 1 [file Data_Sheet_1.docx]

Supplementary Material

**Enhancing the electricity generation and nitrate removal of microbial fuel cell with a novel denitrifying exoelectrogenic strain EB-1**

**Xiaojun Jin ^1, 2^, Fei Guo ^1^, Zhimei Liu ^1, 2^, Yuan Liu ^1 *^, Hong Liu ^1, 3 *^**

*** Correspondence:** Yuan Liu: [liuyuan@cigit.ac.cn](mailto:liuyuan@cigit.ac.cn); Hong Liu: [liuhong@cigit.ac.cn](mailto:liuhong@cigit.ac.cn)

## Supplementary Figures


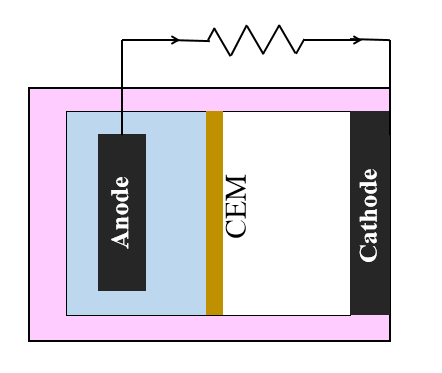


**Supplementary Figure S1. The schematic of the air-cathode MFC**


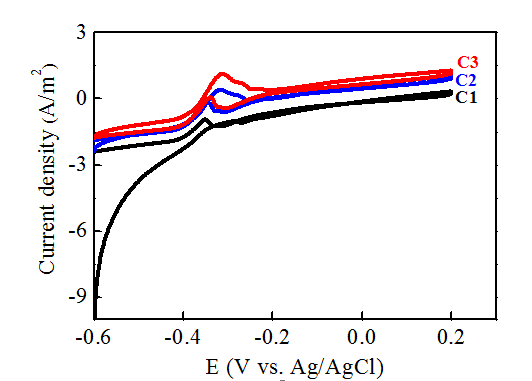


**Supplementary Figure S2. Three circles of CV at initial voltage increasing stage when the anolyte was replaced with fresh medium in few minutes.**


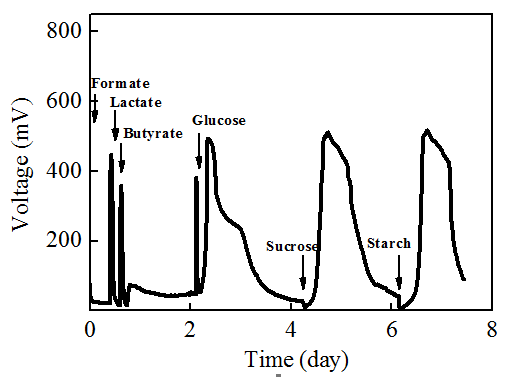


**Supplementary Figure S3. Voltage outputs of MFCs by strain EB-1with different carbon sources (COD=500 mg/L).**

## Supplementary Tables

**Supplementary Table S1. Nitrogen detection of the end-products of MFCs with different initial NO_3_^-^-N concentrations.**

| Initial NO_3_^-^-N concentrations  (mg L^-1^) | Residual NO_3_^-^-N  (mg L^-1^) | Product NO_2_^-^-N  (mg L^-1^) | Product NH_4_^+^-N  (mg L^-1^) |
| --- | --- | --- | --- |
| 22.3±0.5 | 1.27±0.31 | 0.07±0.01 | ND |
| 52.5±1.3 | 1.15±0.16 | 0.07±0.01 | ND |
| 105.4±3.5 | 2.08±0.54 | 0.40±0.02 | ND |
| 222.5±5.1 | 41.34±4.34 | 0.81±0.04 | ND |

ND: the concentration of samples under the limit of detection
